# Gender as an independent prognostic factor in small-cell lung cancer: Inha Lung Cancer Cohort study using propensity score matching

**DOI:** 10.1371/journal.pone.0208492

**Published:** 2018-12-11

**Authors:** Jun Hyeok Lim, Jeong-Seon Ryu, Jae Hoon Kim, Hyun-Jung Kim, DaeHyung Lee

**Affiliations:** 1 Department of Internal Medicine, Inha University Hospital, Incheon, Republic of Korea; 2 Inha University School of Medicine, Incheon, Republic of Korea; 3 Respiratory Public Medical Center, Inha University Hospital, Incheon, Republic of Korea; University of Cincinnati College of Medicine, UNITED STATES

## Abstract

**Introduction:**

The prognostic relevance of gender is undetermined in patients with small-cell lung cancer (SCLC). Therefore, we investigated whether gender is a prognostic factor in a SCLC cohort after controlling for confounding factors.

**Materials and methods:**

Fifteen prognostic factors were classified into four groups (patient, stage migration, tumor, and treatment). The prognostic relevance of gender was evaluated using propensity score matching, Cox proportional hazards regression, and stepwise fashion adjustments.

**Results:**

Of 591 patients with SCLC, 88 were women (14.9%). Women were more likely than men to have no history of smoking (48.9% vs. 2.0%, P *<* 0.001) and limited disease (48.9% vs. 37.8%, *P* = 0.050). Women had less progressive disease in M stage than men (52.3% vs. 62.8%, *P* = 0.031). Women had better survival than men in the entire cohort (median survival times [MSTs] and 95% confidence intervals [CIs]: 9.7 months and 7.8–11.6 for women, 8.0 months and 7.0–8.9 for men, log-rank *P* = 0.034) and in the matched cohort (MSTs and 95% CIs: 8.8 months and 5.8–11.8 for women, 5.9 months and 4.5–7.4 for men, log-rank *P* = 0.013). Female gender was a prognostic factor predicting better survival, even after stepwise and full adjustment with all prognostic variables (adjusted hazard ratios and 95% CIs: 0.51 and 0.34–0.77, *P* = 0.001 for entire cohort, 0.42 and 0.24–0.75, *P* = 0.003 for matched cohort).

**Conclusions:**

Our results confirmed that gender is an independent prognostic factor in patients with SCLC.

## Introduction

Small-cell lung cancer (SCLC) accounts for approximately 13% of all lung cancers and is clinically distinguished by a high occurrence in smokers, early micro-metastasis, and high response rate to chemotherapy [1]. Among patients with SCLC, the proportion of affected women has steadily increased over several decades in the United States, from 27% in 1973 to 50% in recent years [2]. Because SCLC is associated with smoking, researchers have assumed that its prevalence increases incrementally with the proportion of female smokers. However, the prevalence of SCLC varies by geography and remains 10–20% in Asian countries, including Korea [3–5].

Several previous studies have explored whether gender has prognostic significance in patients with SCLC. Although some studies report that gender is a prognostic factor in SCLC [6–10], others report that gender has no prognostic significance [4, 11–14]. Because the survival of cancer patients is affected by a variety of clinical factors related to the patient, stage migration, tumor, and treatment, these confounders should be considered when evaluating the prognostic relevance of gender. However, none of these previous studies adequately controlled for these clinical factors.

We conducted a prospective cohort study to investigate the prognostic relevance of gender in patients with SCLC using Cox proportional hazards regression with stepwise adjustments and propensity score matching (PSM).

## Materials and methods

### Study population

A total of 657 patients histologically diagnosed with SCLC between January 2001 and December 2016 at Inha University Hospital (Incheon, Republic of Korea) were initially considered for this study (S1 Fig). To maintain high-quality of information, patients who did not undergo staging workup (n = 21), were lost to follow up after diagnosis (n = 17), or were diagnosed at other hospitals (n = 16) were excluded. In addition, 12 patients were excluded due to insufficient data. Staging workup was performed for all patients, who were fully followed up with at the hospital. No patient was administered any targeted agents or was enrolled in any clinical trial. All data were prospectively collected from the Inha Lung Cancer Cohort [15]. This study was approved by the Institutional Review Board of Inha University Hospital, which waived the requirement of obtaining written informed consent from patients.

### Prognostic variables

The prognostic variables included the following information: age; gender; disease extent; smoking history; family history of lung cancer; occupation by standard of International Agency for Research on Cancer; any weight loss during the 6 months before diagnosis; Eastern Cooperative Oncology Group (ECOG) performance status; serum levels of hematocrit, albumin, lactate dehydrogenase, calcium, and neutrophil-to-lymphocyte ratio (NLR) at diagnosis; and whether positron emission tomography (PET) scans were performed [16]. Disease extent was categorized as limited or extensive according to Veterans Administration Lung Study Group classification [17]. M stage was recorded according to TNM classification (8^th^ edition) [18]. Patients with extrathoracic metastasis were subdivided into two groups: single extrathoracic metastasis and multiple extrathoracic metastasis. Treatment received by a patient was classified as “curative” if it consisted of surgical resection or more than two cycles of platinum-based chemotherapy and “palliative” if it consisted of one cycle of chemotherapy or palliative radiotherapy.

### Survival measurements

Overall survival was calculated from the time of diagnosis to the time of the last follow up or death as a result of any cause. The median follow-up time was 11.3 months (95% CIs: 2.7–37.1). Death was observed in 537 patients (90.9%), 526 of whom died in our hospital. Only nine patients died of causes other than lung cancer. For 11 patients who could not be contacted after hospital discharge, information on survival was collected from the Korean Ministry of Security and Public Administration.

### Statistical analysis

Prognostic variables between male and female patients were compared using independent samples *t*-tests for continuous variables and Chi-square or Fisher’s exact tests for categorical variables.

The 15 prognostic variables were classified into four groups: patient, stage migration, tumor, and treatment. Patient-related variables were divided into two subgroups: basic and tumor burden. Basic-related variables included age, smoking history, family history of lung cancer, and occupation. Tumor burden-related variables included ECOG performance status, weight loss, hematocrit, albumin, lactate dehydrogenase, calcium, and NLR. The stage migration-related variable was whether PET scans had been taken. Tumor-related variables were disease extent and M stage. Finally, treatment-related variables consisted of curative treatment, palliative treatment, and no treatment.

To minimize the covariate imbalance in baseline characteristics between genders, PSM was performed. Propensity scores were estimated by multiple logistic regression. The 2:1 matching was performed using nearest-neighbor matching with a caliper width equal to 0.1 standard deviations.

The effect of gender on survival was estimated by the Kaplan-Meier method and log-rank testing. Hazard ratios (HRs) and 95% confidence intervals (CIs) were estimated by Cox proportional hazards regression within the entire cohort and within the matched cohort. HRs were calculated for the unadjusted model and then for the adjusted models by stepwise adding each prognostic group [15]. The 15 prognostic variables were adjusted in the final model. The accuracy of the Cox models was evaluated by Harrell’s c-index. All statistical tests were two-sided, and *P value*s < 0.05 were considered statistically significant.

Analyses were performed using a statistical software package (SPSS version 19.0, SPSS, Chicago, IL).

## Results

### Patient characteristics in the entire cohort

The baseline characteristics of 591 patients with SCLC by gender are shown in Table 1. There were 88 female patients (14.9%). The median age of patients was 68 years, with women being significantly older than men (70 years vs. 67 years, *P* = 0.030). Fifty-three (8.9%) patients had no history of smoking. The proportion of never-smokers among women was significantly larger than that among men (48.9% vs. 2.0%, P *<* 0.001). Women were more likely to present at diagnosis with limited disease and less progressive disease in M stage than men (51.1% vs. 62.2%, *P* = 0.050; 52.3% vs. 62.8%, *P* = 0.031, respectively). No significant differences were observed between men and women in treatment; family history of lung cancer; ECOG performance status; weight loss; serum levels of albumin, lactate dehydrogenase, calcium, or NLR; or the proportion of patients undergoing PET.

**Table 1:** Characteristics of small-cell lung cancer patients in entire and matched cohorts.

	Entire cohort (n = 591)			Matched cohort (n = 150)		
Variables	Male (n = 503)	Female (n = 88)	*P* value	Male (n = 97)	Female (n = 53)	*P* value
Median age, years (range)	67 (35–93)	70 (40–89)	0.030	72 (35–87)	70 (40–89)	0.592
Smoking history, n (%)						
Never	10 (2.0)	43 (48.9)	<0.001	9 (9.3)	9 (17.0)	0.165
Ever	493 (98.0)	45 (51.1)		88 (90.7)	44 (83.0)	
Family history, n (%)						
Yes	26 (5.4)	3 (3.5)	0.601^b^	7 (7.7)	3 (5.9)	1.000^b^
No	456 (94.6)	83 (96.5)		84 (92.3)	48 (94.1)	
Occupation^a^, n (%)						
1, 2A	79 (17.1)	3 (3.6)	0.001^b^	1 (1.1)	3 (6.0)	0.135^b^
2B, 3, 4	384 (82.9)	81 (96.4)		87 (98.9)	47 (94.0)	
ECOG performance status, n (%)						
0–1	266 (62.1)	38 (53.5)	0.168	48 (57.1)	21 (47.7)	0.310
≥ 2	162 (37.9)	33 (46.5)		36 (42.9)	23 (52.3)	
Weight loss (%), n (%)						
None to < 5	205 (43.2)	40 (47.1)	0.514	41 (45.1)	24 (47.1)	0.818
≥ 5	269 (56.8)	45 (52.9)		50 (54.9)	27 (52.9)	
Hematocrit^c^, n (%)						
Low^d^	239 (47.5)	58 (65.9)	0.001	60 (61.9)	32 (60.4)	0.859
High	264 (52.5)	30 (34.1)		37 (38.1)	21 (39.6)	
Albumin^c^, n (%)						
Low	278 (55.3)	51 (58.0)	0.640	59 (60.8)	29 (54.7)	0.468
High	225 (44.7)	37 (42.0)		38 (39.2)	24 (45.3)	
Lactate dehydrogenase^c^, n (%)						
Low	186 (49.3)	33 (55.9)	0.346	41 (51.9)	19 (52.8)	0.930
High	191 (50.7)	26 (44.1)		38 (48.1)	17 (47.2)	
Calcium^c^, n (%)						
Low	288 (57.3)	44 (50.0)	0.206	57 (58.8)	26 (49.1)	0.253
High	215 (42.7)	44 (50.0)		40 (41.2)	27 (50.9)	
NLR^c^, n (%)						
Low	252 (50.1)	45 (51.1)	0.858	40 (41.2)	24 (45.3)	0.632
High	251 (49.9)	43 (48.9)		57 (58.8)	29 (54.7)	
PET, n (%)						
Yes	215 (42.7)	41 (46.6)	0.502	39 (40.2)	24 (45.3)	0.547
No	288 (57.3)	47 (53.4)		58 (59.8)	29 (54.7)	
Disease extent^d^, n (%)						
Limited	190 (37.8)	43 (48.9)	0.050	47 (48.5)	25 (47.2)	0.880
Extensive	313 (62.2)	45 (51.1)		50 (51.5)	28 (52.8)	
M stage, n (%)						
M0	187 (37.2)	42 (47.7)	0.031	47 (48.5)	25 (47.2)	0.961
M1a	77 (15.3)	6 (6.8)		6 (6.2)	3 (5.7)	
M1b	64 (12.7)	16 (18.2)		15 (15.5)	10 (18.9)	
M1c	175 (34.8)	24 (27.3)		29 (29.8)	15 (28.2)	
Treatment^e^, n (%)						
Curative	289 (59.8)	50 (61.0)	0.663	44 (46.3)	27 (56.2)	0.337
Palliative	83 (17.2)	11 (13.4)		26 (27.4)	8 (16.7)	
No	111 (23.0)	21 (25.6)		25 (26.3)	13 (27.1)	

^a^ By standard of International Agency for Research on Cancer.

^b^ Fisher's exact test.

^c^ Dichotomized by cutoff of median value.

^d^ Disease extent was categorized as limited or extensive according to Veterans Administration Lung Study Group classification.

^e^ The treatment the patient received was classified as ‘curative treatment’ if they received surgical resection or more than two cycles of platinum-based chemotherapy and ‘palliative treatment’ if they received less than one cycle of chemotherapy or palliative radiotherapy. ECOG, Eastern Cooperative Oncology Group; NLR, neutrophil-to-lymphocyte ratio; PET, positron emission tomography

### Patient characteristics in the matched cohort

Using 2:1 PSM, 53 female patients were matched to 97 male patients. After PSM, no differences between men and women were observed for the following prognostic variables: age (*P* = 0.592), smoking history (*P* = 0.165), occupation (*P* = 0.135), hematocrit (*P* = 0.859), disease extent (*P* = 0.880), and M stage (*P* = 0.961).

### Gender and overall survival in the entire cohort

Most variables related to patient, tumor, and treatment had significant effects on overall survival, excluding smoking history, family history of lung cancer, occupation, and proportion of patients undergoing PET (S1 Table). Never smokers showed a trend toward better survival than smokers, but this was not statistically significant (median survival time [MST]: 10.0 months vs. 8.2 months, log-rank *P* = 0.346). Smoking history was independent prognostic factor when adjusted with age, gender, EGOG performance status, disease extent, and treatment (adjusted HR and 95% CI: 1.52 and 1.01–2.28; Cox *P* = 0.044, data not shown).

**Fig 1:**
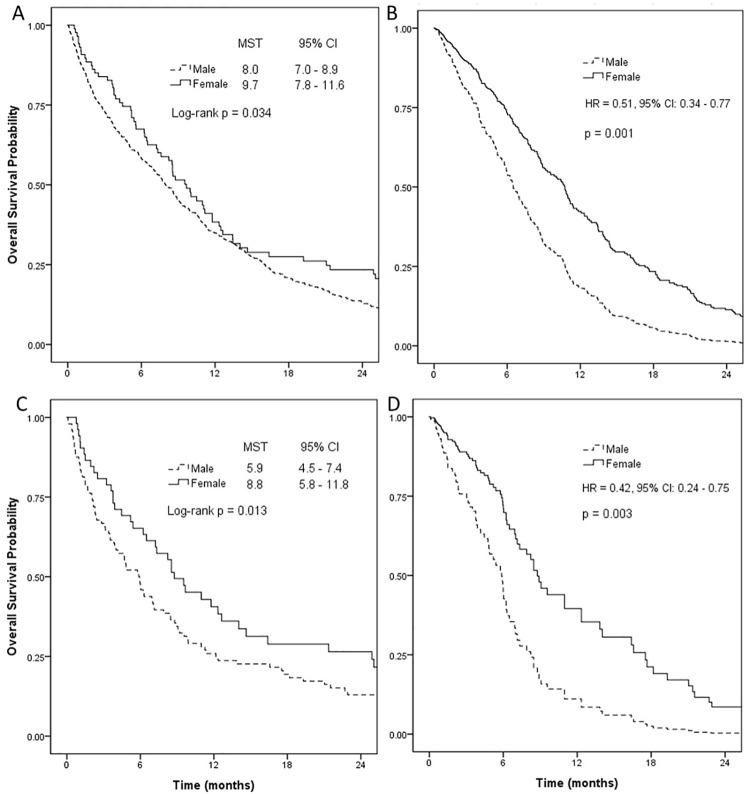
Overall survival of small-cell lung cancer patients by gender. (A) Kaplan-Meier plot and (B) fully adjusted Cox model in entire cohort; (C) Kaplan-Meier plot and (D) fully adjusted Cox model in propensity score matched cohort. MST, median survival time; CI, confidence interval.

Women had significantly better survival than men (MSTs and 95% CIs: 9.7 months and 7.8–11.6 for women, 8.0 months and 7.0–8.9 for men; log-rank *P* = 0.034; Fig 1A). In Cox proportional hazards models, female gender was a consistent and significant prognostic factor predicting better survival when unadjusted and adjusted in a stepwise fashion (unadjusted HR and 95% CI: 0.77 and 0.60–0.98; adjusted HRs and 95% CIs: 0.60 and 0.43–0.83 for basic and 0.46 and 0.31–0.70 for tumor burden as patient-related variables, 0.46 and 0.30–0.69 for the stage migration-related variable, 0.52 and 0.35–0.79 for tumor-related variables, and 0.51 and 0.34–0.77 for the treatment-related variable; Table 2, Fig 1B, and S2 Fig). Harrell’s c-index was 0.79 for the final model. When analyzed in ever smokers, gender was an independent prognostic factor (adjusted HRs and 95% CIs: 0.42 and 0.26–0.69 for the final model, S2 Table).

**Table 2:** Effect of gender on overall survival in entire and matched cohorts: Cox proportional hazard modeling results.

Variables	Entire cohort (n = 591)		Matched cohort (n = 150)	
	HR (95% CI)	*P* value	HR (95% CI)	*P* value
Unadjusted	0.77 (0.60–0.98)	0.035	0.63 (0.44–0.91)	0.014
Plus patient-related				
Basic: age, smoking history, family history, occupation	0.60 (0.43–0.83)	0.002	0.63 (0.42–0.94)	0.024
Tumor burden: ECOG performance status, weight loss, hematocrit, albumin, lactate dehydrogenase, calcium, NLR	0.46 (0.31–0.70)	<0.001	0.54 (0.33–0.90)	0.018
Plus stage migration-related				
PET	0.46 (0.30–0.69)	<0.001	0.53 (0.32–0.88)	0.014
Plus tumor-related				
Disease extent, M stage	0.52 (0.35–0.79)	0.002	0.50 (0.30–0.84)	0.009
Plus treatment-related (final model)	0.51 (0.34–0.77)	0.001	0.42 (0.24–0.75)	0.003

HR, hazard ratio; CI, confidence interval; ECOG, Eastern Cooperative Oncology Group; NLR, neutrophil-to-lymphocyte ratio; PET, positron emission tomography

### Gender and overall survival in the matched cohort

Women had significantly better survival than men (MSTs and 95% CIs, 8.8 months and 5.8–11.8 for women, 5.9 months and 4.5–7.4 for men; log-rank *P* = 0.013; Fig 1C). As shown in Table 2, the impact of female gender as a favorable prognostic factor was observed in every stepwise adjustment and in the final model (unadjusted HR and 95% CI: 0.63 and 0.44–0.91, adjusted HRs and 95% CIs: 0.63 and 0.42–0.94 for basic and 0.54 and 0.33–0.90 for tumor burden as patient-related variables, 0.53 and 0.32–0.88 for the stage migration-related variable, 0.50 and 0.30–0.84 for tumor-related variables, and 0.42 and 0.24–0.75 for the treatment-related variable; Fig 1D, and S3 Fig). Harrell’s c-index was 0.78 for the final model.

## Discussion

To our knowledge, this is the first prospective cohort study demonstrating that gender is an independent prognostic factor in patients with SCLC. Fifteen confounding variables were considered, and Cox proportional hazards models with stepwise adjustment and PSM were used. Analyses of the entire cohort and the matched cohort provided consistent results indicating more favorable survival in women with SCLC.

Significant differences between men and women with SCLC were observed for clinical variables including age, smoking history, disease extent, and M stage. Tumor-related variables, including disease extent and M stage, are known to be strong prognostic factors. Although the small number of never-smokers among patients with SCLC is a significant hurdle for evaluating prognostic effects, previous studies report that smoking history has only a marginal effect on survival [4, 11]. Therefore, robust methods for controlling confounders or bias is needed to verify the prognostic effect of gender.

Most previous studies used regression adjustments such as Cox proportional hazard models for evaluating the prognostic relevance of gender [4, 6–14]. PSM was developed to eliminate selection bias and potential baseline differences between comparison groups [19]. PSM has the advantage of eliminating residual bias that may remain after regression adjustment. Moreover, PSM followed by regression adjustment is superior to regression adjustment alone [20]. Therefore, in the present study, we analyzed the prognostic effect of gender with Cox proportional hazard models in a matched cohort.

The prognostic relevance of gender was maintained in every step of adjustment in this study, indicating the robustness of gender as a prognostic factor. Moreover, we found that the prognostic impact of gender gradually increased as the adjustment steps proceeded. The risk of death decreased gradually from the unadjusted model to the final model in the entire cohort (from 23% to 49% reduction) and in the matched cohort (from 37% to 58% reduction). Contrary to NSCLC, SCLC studies have rarely incorporated advanced technologies like NGS, and within those studies it appears that there is no gender difference in somatic mutation [4, 21]. Further investigations of genetic, hormonal, and metabolic factors are needed to explain the difference in survival between genders.

This study has several limitations. First, the dose intensity of chemotherapy and accompanied toxicities, delay in diagnosis or treatment, and socioeconomic status of patients could affect patient survival. Although these factors were not considered in this study, a total of 15 clinical factors related to the patient, tumor, stage migration, and treatment were prospectively acquired and systematically adjusted in this analysis. Second, because this study was conducted using a single cohort, its results may be limited to this population and must be validated with other cohorts. Nevertheless, the proportions of women and smokers among patients with SCLC observed in this study are similar to those reported by other Korean studies [4, 5]. Third, overall survival may be a suboptimal measurement compared with cancer-specific survival. However, deaths and related causes were stringently ascertained in the cohort, with only 9 of the 526 reported deaths being due to non-cancer causes. Fourth, the potential for bias could be concerned when propensity score matching is used in case of a rare exposure and small sample size. Propensity score weighting (inverse probability of treatment weights) was suggested in such situation [22]. When evaluated with propensity score weighting, gender was maintained as an independent prognostic factor (adjusted HRs and 95% CIs: 0.53 and 0.34–0.82, *P* = 0.004 for the final model) (data not shown).

In conclusion, we confirmed that gender is an independent prognostic factor in patients with SCLC. Further studies are required to verify this gender difference.

## Supporting information

S1 Fig Flow chart for enrollment and classification of patients. (TIF)

S2 Fig Overall survival of small-cell lung cancer patients by gender in entire cohort. (A) unadjusted model, (B) stepwise models adjusted with basic, (C) tumor burden, (D) stage migration, (E) tumor. HR; hazard ratio, CI; confidence interval.
(TIF)

S3 Fig Overall survival of small-cell lung cancer patients by gender in propensity score matched cohort. (A) unadjusted model, (B) stepwise models adjusted with basic, (C) tumor burden, (D) stage migration, (E) tumor. HR; hazard ratio, CI; confidence interval.
(TIF)

S1 Table Prognostic variables and overall survival of small-cell lung cancer patients in entire and propensity score matched cohort. (DOCX)

S2 Table Effect of gender on overall survival in ever-smokers: Cox proportional hazard modeling results. (DOCX)

## References

[1] Howlader N, Noone A, Krapcho M, Miller D, Bishop K, Kosary C, et al. SEER Cancer Statistics Review, 1975–2014, National Cancer Institute. Bethesda, MD; 2017 [cited 2017 Sep 2]. https://seer.cancer.gov/csr/1975_2014/.

[2] Govindan R, Page N, Morgensztern D, Read W, Tierney R, Vlahiotis A, et al Changing epidemiology of small-cell lung cancer in the United States over the last 30 years: analysis of the surveillance, epidemiologic, and end results database. J Clin Oncol. 2006; 24(28):4539–4544. 10.1200/JCO.2005.04.4859 . 17008692 10.1200/JCO.2005.04.4859

[3] Cheng T-YD, Cramb SM, Baade PD, Youlden DR, Nwogu C, Reid ME. The international epidemiology of lung cancer: latest trends, disparities, and tumor characteristics. J Thorac Oncol. 2016; 11(10):1653–1671. 10.1016/j.jtho.2016.05.021 27364315 10.1016/j.jtho.2016.05.021 PMC5512876

[4] Sun J-M, Choi Y-L, Ji J, Ahn J, Kim K-M, Han J, et al Small-cell lung cancer detection in never-smokers: clinical characteristics and multigene mutation profiling using targeted next-generation sequencing. Ann Oncol. 2014; 26(1):161–6. 10.1093/annonc/mdu504 25355724 10.1093/annonc/mdu504

[5] Shin A, Oh C-M, Kim B-W, Woo H, Won Y-J, Lee J-S. Lung cancer epidemiology in Korea. Cancer Res Treat. 2017; 49(3):616 10.4143/crt.2016.178 27669705 10.4143/crt.2016.178 PMC5512360

[6] Johnson BE, Steinberg SM, Phelps R, Edison M, Veach SR, Ihde DC. Female patients with small cell lung cancer live longer than male patients. Am J Med. 1988; 85(2):194–196. 2840825 10.1016/s0002-9343(88)80341-3

[7] Wolf M, Holle R, Hans K, Drings P, Havemann K. Analysis of prognostic factors in 766 patients with small cell lung cancer (SCLC): the role of sex as a predictor for survival. Br J Cancer. 1991; 63(6):986–992. 1648949 10.1038/bjc.1991.215 PMC1972562

[8] Sagman U, Maki E, Evans W, Warr D, Shepherd F, Sculier J, et al Small-cell carcinoma of the lung: derivation of a prognostic staging system. J Clin Oncol. 1991; 9(9):1639–1649. 10.1200/JCO.1991.9.9.1639 1651996 10.1200/JCO.1991.9.9.1639

[9] Paesmans M, Sculier J-P, Lecomte J, Thiriaux J, Libert P, Sergysels R, et al Prognostic factors for patients with small cell lung carcinoma: analysis of a series of 763 patients included in 4 consecutive prospective trials with a minimum follow-up of 5 years. Cancer. 2000; 89(3):523–533. 10931451 10.1002/1097-0142(20000801)89:3<523::aid-cncr7>3.0.co;2-6

[10] Ou S-HI, Ziogas A, Zell JA. Prognostic factors for survival in extensive stage small cell lung cancer (ED-SCLC): the importance of smoking history, socioeconomic and marital statuses, and ethnicity. J Thorac Oncol. 2009; 4(1):37–43. 10.1097/JTO.0b013e31819140fb 19096304 10.1097/JTO.0b013e31819140fb

[11] McCracken JD, Janaki LM, Crowley JJ, Taylor SA, Giri P, Weiss GB, et al Concurrent chemotherapy/radiotherapy for limited small-cell lung carcinoma: a Southwest Oncology Group Study. J Clin Oncol. 1990; 8(5):892–898. 10.1200/JCO.1990.8.5.892 2159055 10.1200/JCO.1990.8.5.892

[12] Lassen U, Osterlind K, Hansen M, Dombernowsky P, Bergman B, Hansen HH. Long-term survival in small-cell lung cancer: posttreatment characteristics in patients surviving 5 to 18+ years—an analysis of 1,714 consecutive patients. J Clin Oncol. 1995; 13(5):1215–1220. 10.1200/JCO.1995.13.5.1215 7738624 10.1200/JCO.1995.13.5.1215

[13] Quoix E, Purohit A, Faller-Beau M, Moreau L, Oster J, Pauli G. Comparative prognostic value of lactate dehydrogenase and neuron-specific enolase in small-cell lung cancer patients treated with platinum-based chemotherapy. Lung cancer. 2000; 30(2):127–134. 11086206 10.1016/s0169-5002(00)00131-8

[14] Mennecier B, Lebitasy M-P, Moreau L, Hedelin G, Purohit A, Galichet C, et al Women and small cell lung cancer: social characteristics, medical history, management and survival: a retrospective study of all the male and female cases diagnosed in Bas-Rhin (Eastern France) between 1981 and 1994. Lung Cancer. 2003; 42(2):141–152. 14568681 10.1016/s0169-5002(03)00284-8

[15] Ryu J-S, Ryu HJ, Lee S-N, Memon A, Lee S-K, Nam H-S, et al Prognostic impact of minimal pleural effusion in non–small-cell lung cancer. J Clin Oncol. 2014; 32(9):960–967. 10.1200/JCO.2013.50.5453 24550423 10.1200/JCO.2013.50.5453

[16] Dinan MA, Curtis LH, Carpenter WR, Biddle AK, Abernethy AP, Patz EF Jr, et al Stage migration, selection bias, and survival associated with the adoption of positron emission tomography among medicare beneficiaries with non-small-cell lung cancer, 1998–2003. J Clin Oncol. 2012; 30(22):2725–30. 10.1200/JCO.2011.40.4392 22753917 10.1200/JCO.2011.40.4392

[17] Zelen M. Keynote address on biostatistics and data retrieval. Cancer Chemother Rep 3. 1973; 4(2):31–42. 4580860

[18] Nicholson AG, Chansky K, Crowley J, Beyruti R, Kubota K, Turrisi A, et al The International Association for the Study of Lung Cancer Staging Project: proposals for the revision of the clinical and pathologic staging of small cell lung cancer in the forthcoming eighth edition of the TNM classification for lung cancer. J Thorac Oncol. 2016; 11(3):300–311. 10.1016/j.jtho.2015.10.008 26723244 10.1016/j.jtho.2015.10.008

[19] Rosenbaum PR, Rubin DB. Reducing bias in observational studies using subclassification on the propensity score. J Am Stat Assoc. 1984; 79(387):516–524.

[20] Rubin DB, Thomas N. Combining propensity score matching with additional adjustments for prognostic covariates. J Am Stat Assoc. 2000; 95(450):573–585.

[21] Peifer M, Fernández-Cuesta L, Sos ML, George J, Seidel D, Kasper LH, et al Integrative genome analyses identify key somatic driver mutations of small-cell lung cancer. Nat Genet. 2012; 44(10):1104 10.1038/ng.2396 22941188 10.1038/ng.2396 PMC4915822

[22] Hajage D, Tubach F, Steg PG, Bhatt DL, De Rycke Y. On the use of propensity scores in case of rare exposure. BMC Med Res Methodol. 2016;16(1):38. 27036963 10.1186/s12874-016-0135-1 PMC4815252

