# Supplementary figures and images for: Gender as an independent prognostic factor in small-cell lung cancer: Inha Lung Cancer Cohort study using propensity score matching
Source: PLoS One. 2018 Dec 11;13(12):e0208492. doi: 10.1371/journal.pone.0208492 (PMC6289417; doi:10.1371/journal.pone.0208492)

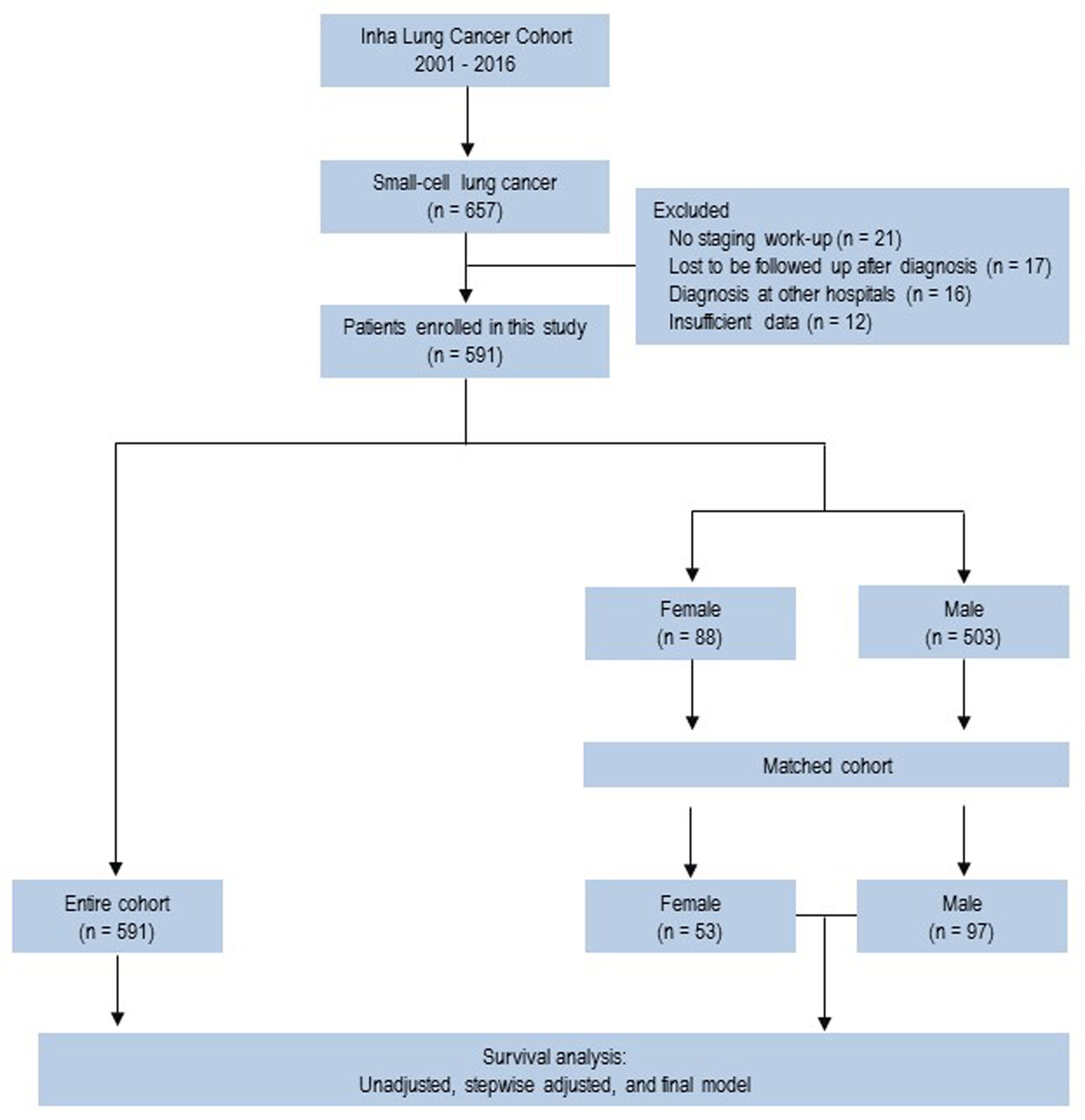

Supplement: S1 Fig — (TIF) [file pone.0208492.s001.tif]

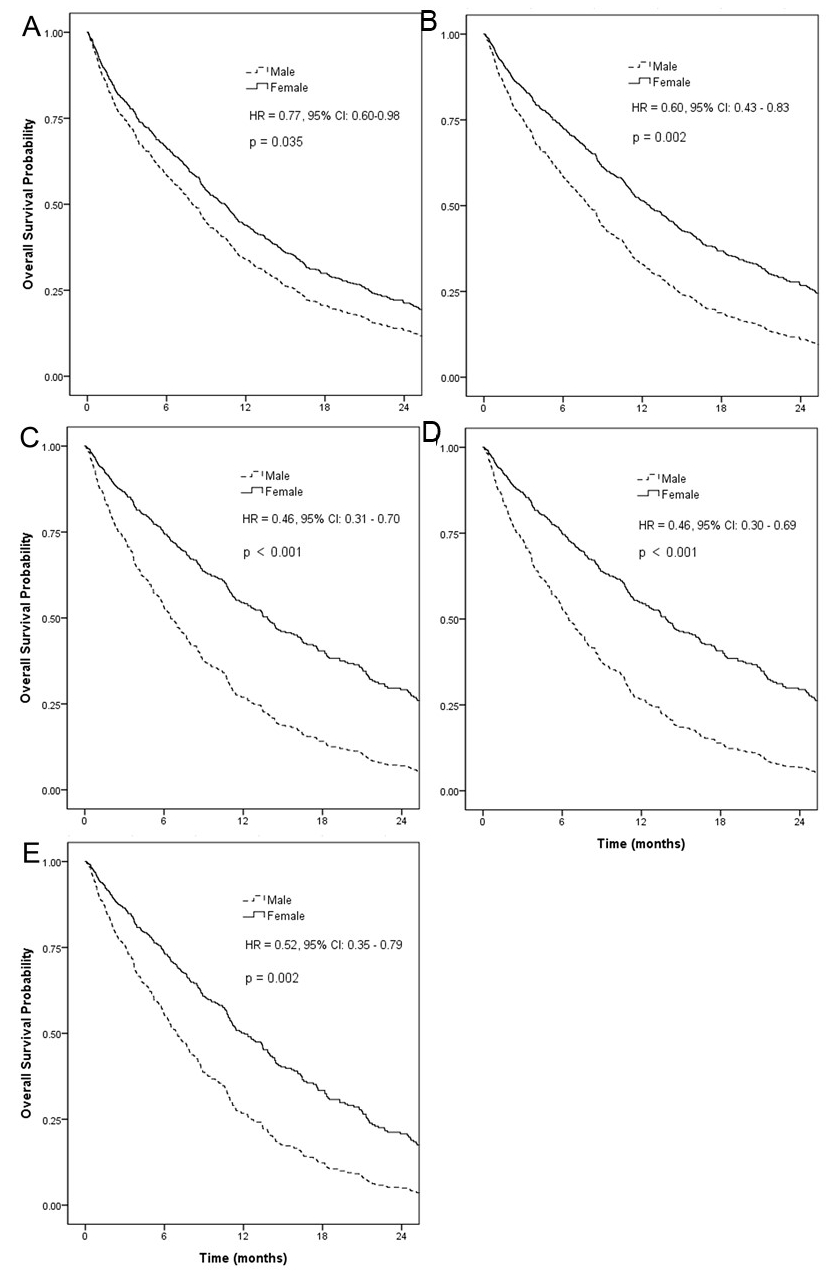

Supplement: S2 Fig — (A) unadjusted model, (B) stepwise models adjusted with basic, (C) tumor burden, (D) stage migration, (E) tumor. HR; hazard ratio, CI; confidence interval. (TIF) [file pone.0208492.s002.tif]

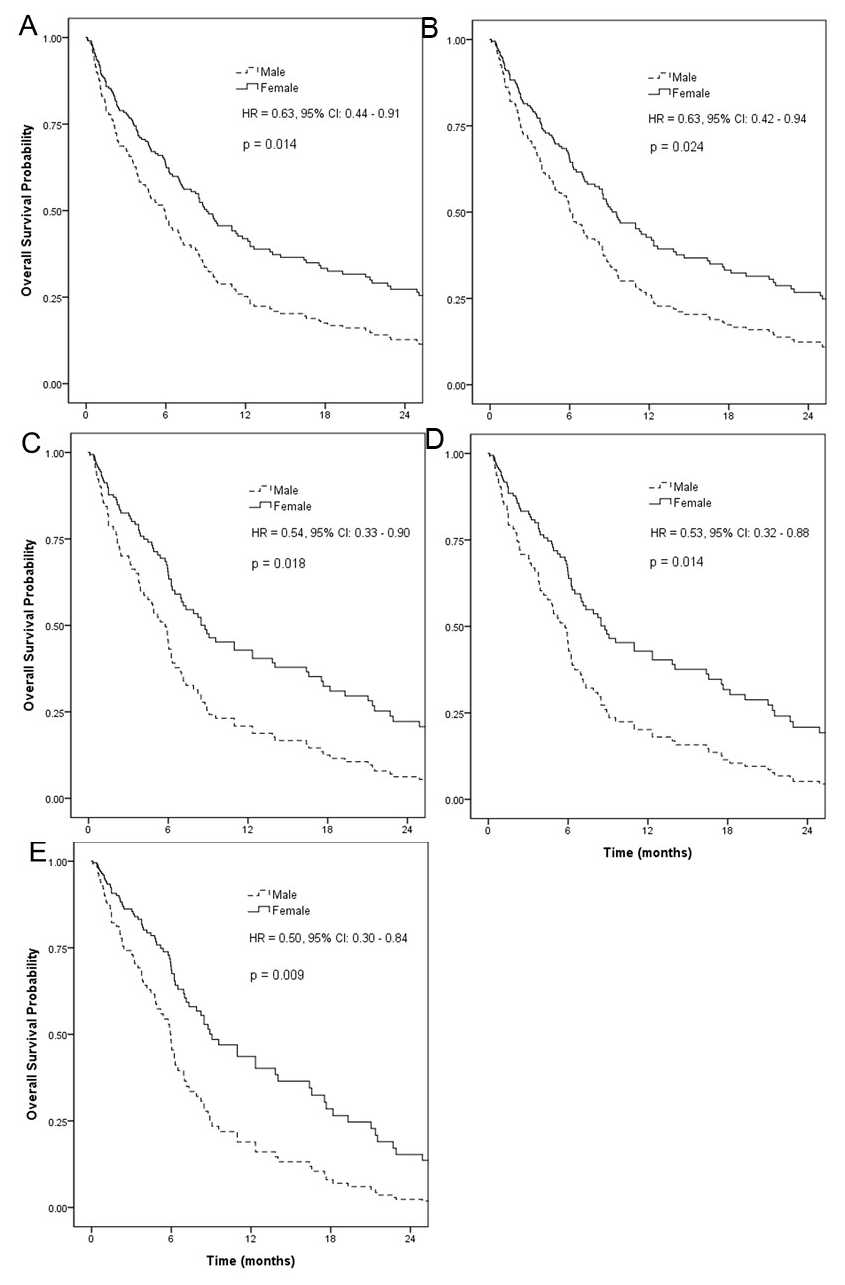

Supplement: S3 Fig — (A) unadjusted model, (B) stepwise models adjusted with basic, (C) tumor burden, (D) stage migration, (E) tumor. HR; hazard ratio, CI; confidence interval. (TIF) [file pone.0208492.s003.tif]
