# Supplementary material for: Gender as an independent prognostic factor in small-cell lung cancer: Inha Lung Cancer Cohort study using propensity score matching
Source: PLoS One. 2018 Dec 11;13(12):e0208492. doi: 10.1371/journal.pone.0208492 (PMC6289417; doi:10.1371/journal.pone.0208492)
Supplement: S1 Table — (DOCX) [file pone.0208492.s004.docx]

**S1 Table. Prognostic variables and overall survival of small-cell lung cancer patients in entire and matched cohorts.**

|  | Entire cohort | | |  | Matched cohort | | |
| --- | --- | --- | --- | --- | --- | --- | --- |
| Variables | MST (months) | HR | *P* value |  | MST (months) | HR | *P* value |
| Gender |  |  | 0.034 |  |  |  | 0.013 |
| Male | 8.0 (7.0-8.9) | 1 |  |  | 5.9 (4.5-7.4) | 1 |  |
| Female | 9.7 (7.8-11.6) | 0.77 (0.60-0.98) |  |  | 8.8 (5.8-11.8) | 0.63 (0.44-0.91) |  |
| Age per year |  | 1.04 (1.03-1.05) | ＜0.001 |  |  | 1.03 (1.01-1.05) | 0.011 |
| Smoking history |  |  | 0.346 |  |  |  | 0.724 |
| Never | 10.0 (6.2-13.8) | 1 |  |  | 7.0 (0.0-19.6) | 1 |  |
| Ever | 8.2 (7.4-9.1) | 1.16 (0.85-1.57) |  |  | 6.5 (4.9-8.1) | 1.10 (0.64-1.89) |  |
| Family history |  |  | 0.092 |  |  |  | 0.604 |
| Yes | 14.7 (7.8-21.6) | 1 |  |  | 11.0 (1.6-20.3) | 1 |  |
| No | 8.1 (7.3-9.0) | 1.41 (0.94-2.11) |  |  | 6.3 (5.1-7.5) | 1.20 (0.61-2.37) |  |
| Occupation^a^ |  |  | 0.174 |  |  |  | 0.458 |
| 1, 2A | 10.7 (8.9-12.6) | 1 |  |  | 9.5 (0.0-19.4) | 1 |  |
| 2B, 3, 4 | 8.1 (7.1-9.1) | 1.19 (0.93-1.52) |  |  | 7.0 (5.1-8.9) | 1.46 (0.54-3.96) |  |
| ECOG performance status |  |  | ＜0.001 |  |  |  | 0.006 |
| 0-1 | 11.2 (10.0-12.3) | 1 |  |  | 9.8 (6.5-13.1) | 1 |  |
| ≥ 2 | 3.8 (2.5-5.1) | 1.91 (1.58-2.32) |  |  | 4.5 (3.1-5.9) | 1.68 (1.2-2.4) |  |
| Weight loss (%) |  |  | ＜0.001 |  |  |  | 0.289 |
| None to < 5 | 10.5 (9.2-11.8) | 1 |  |  | 8.2 (6.7-9.7) | 1 |  |
| ≥ 5 | 5.2 (3.8-6.6) | 1.56 (1.30-1.85) |  |  | 5.4 (3.1-7.7) | 1.21 (0.85-1.71) |  |
| Hematocrit^b^ |  |  | ＜0.001 |  |  |  | 0.045 |
| Low | 6.3 (4.9-7.7) | 1 |  |  | 5.2 (3.3-7.2) | 1 |  |
| High | 10.7 (8.7-12.7) | 0.68 (0.57-0.80) |  |  | 8.9 (5.7-12.1) | 0.70 (0.49-0.99) |  |
| Albumin^b^ |  |  | ＜0.001 |  |  |  | 0.029 |
| Low | 5.8 (4.7-6.8) | 1 |  |  | 5.2 (3.1-7.3) | 1 |  |
| High | 11.4 (9.7-13.2) | 0.62 (0.52-0.73) |  |  | 8.8 (7.0-10.5) | 0.68 (0.48-0.96) |  |
| Lactate dehydrogenase^b^ |  |  | ＜0.001 |  |  |  | 0.020 |
| Low | 10.0 (8.5-11.6) | 1 |  |  | 7.1 (4.8-9.5) | 1 |  |
| High | 5.0 (4.0-6.1) | 1.53 (1.26-1.86) |  |  | 4.7 (3.3-6.0) | 1.57 (1.07-2.30) |  |
| Calcium^b^ |  |  | 0.001 |  |  |  | 0.368 |
| Low | 7.0 (5.8-8.2) | 1 |  |  | 5.9 (3.9-7.8) | 1 |  |
| High | 10.4 (8.9-11.9) | 0.74 (0.63-0.88) |  |  | 7.3 (5.2-9.5) | 0.86 (0.61-1.20) |  |
| NLR^b^ |  |  | ＜0.001 |  |  |  | 0.110 |
| Low | 11.0 (9.1-12.8) | 1 |  |  | 8.2 (6.0-10.5) | 1 |  |
| High | 5.4 (4.2-6.7) | 1.51 (1.27-1.79) |  |  | 4.8 (3.1-6.6) | 1.32 (0.94-1.87) |  |
| PET |  |  | 0.052 |  |  |  | 0.253 |
| Yes | 9.3 (7.9-10.6) | 1 |  |  | 7.3 (5.2-9.5) | 1 |  |
| No | 7.3 (5.9-8.6) | 1.19 (1.00-1.41) |  |  | 6.0 (3.6-8.4) | 1.23 (0.86-1.75) |  |
| Disease extent^c^ |  |  | ＜0.001 |  |  |  | 0.001 |
| Limited | 13.3 (10.7-16.0) | 1 |  |  | 8.5 (4.6-12.4) | 1 |  |
| Extensive | 5.9 (4.8-7.1) | 2.26 (1.88-2.71) |  |  | 5.2 (3.3-7.1) | 1.85 (1.30-2.64) |  |
| M stage |  |  | ＜0.001 |  |  |  | < 0.001 |
| M0 | 13.3 (10.5-16.2) | 1 |  |  | 8.5 (4.6-12.4) | 1 |  |
| M1a | 8.0 (5.2-10.8) | 1.65 (1.27-2.16) |  |  | 5.9 (0.0-13.3) | 1.04 (0.49-2.18) |  |
| M1b | 8.1 (6.3-10.0) | 1.98 (1.51-2.61) |  |  | 8.5 (6.3-10.6) | 1.54 (0.94-2.54) |  |
| M1c | 4.5 (3.4-5.5) | 3.02 (2.44-3.74) |  |  | 4.2 (2.4-6.0) | 2.70 (1.76-4.14) |  |
| Treatment^d^ |  |  | ＜0.001 |  |  |  | < 0.001 |
| Curative | 12.7 (11.3-14.1) | 1 |  |  | 12.2 (8.6-15.7) | 1 |  |
| Palliative | 3.0 (2.1-4.0) | 4.10 (3.20-5.25) |  |  | 2.2 (0.0-4.4) | 3.56 (2.27-5.58) |  |
| No | 1.5 (1.2-1.8) | 6.05 (4.85-7.55) |  |  | 2.3 (2.0-2.7) | 4.07 (2.64-6.27) |  |

^a^ By standard of International Agency for Research on Cancer.

^b^ Dichotomized by median value.

^c^ Disease extent was categorized as limited or extensive according to Veterans Administration Lung Study Group classification.

^d^ The treatment the patient received was classified as ‘curative treatment’ if they received surgical resection or more than two cycles of platinum-based chemotherapy and ‘palliative treatment’ if they received less than one cycle of chemotherapy or palliative radiotherapy.

MST, median survival time; HR, hazard ratio; ECOG, Eastern Cooperative Oncology Group; NLR, neutrophil-to-lymphocyte ratio; PET, positron emission tomography
