# Supplementary material for: Gender as an independent prognostic factor in small-cell lung cancer: Inha Lung Cancer Cohort study using propensity score matching
Source: PLoS One. 2018 Dec 11;13(12):e0208492. doi: 10.1371/journal.pone.0208492 (PMC6289417; doi:10.1371/journal.pone.0208492)
Supplement: S2 Table — (DOCX) [file pone.0208492.s005.docx]

**S2 Table. Effect of gender on overall survival in ever-smokers (n = 538): Cox proportional hazard modeling results.**

| Variables | HR (95% CI) | *P* value |
| --- | --- | --- |
| Unadjusted | 0.75 (0.54-1.06) | 0.103 |
| Plus patient-related |  |  |
| Basic: age, family history, occupation | 0.61 (0.42-0.87) | 0.007 |
| Tumor burden: ECOG performance status, weight loss, hematocrit, albumin, lactate dehydrogenase, calcium, NLR | 0.46 (0.29-0.74) | 0.001 |
| Plus stage migration-related |  |  |
| PET | 0.46 (0.29-0.73) | 0.001 |
| Plus tumor-related |  |  |
| Disease extent, M stage | 0.50 (0.31-0.80) | 0.004 |
| Plus treatment-related (final model) | 0.42 (0.26-0.69) | 0.001 |

HR, hazard ratio; CI, confidence interval; ECOG, Eastern Cooperative Oncology Group; NLR, neutrophil-to-lymphocyte ratio; PET, positron emission tomography
